# The New Seamen's Hospital, Cardiff

**Published:** 1900-11-10

**Authors:** 


### Editor's Letter-Box.

[Our Correspondents are reminded that prolixity is a great bar to publication, and that brevity of style and conciseness of statement greatly facilitate early insertion.]

### THE NEW SEAMEN'S HOSPITAL, CARDIFF.

A correspondent, writing from Cardiff in regard to the new Seamen's Hospital which is being prepared for at Cardiff, says: I cannot help thinking that a very serious mistake is being made with regard to the site. The old Seamen's Hospital is a wooden ship, moored close to the entrance of the old canal, Cardiff. The site chosen (apparently) by the late Marquis of Bute for the new permanent hospital is on the foreshore — and I may say that the foreshore here is mainly composed of clay soil. With every precaution for safety, I cannot help thinking that (1) the subsoil is unsuitable; (2) that the distance from the docks is too great, and (3) that the cost of filling up is more than is necessary. As the hospital is solely intended for the use of sailors, it would be much better to locate it in the vicinity of the ships.

